# Risk, Trust, and Emotion in Online Pharmacy Medication Purchases: Multimethod Approach Incorporating Customer Self-Reports, Facial Expressions, and Neural Activation

**DOI:** 10.2196/48850

**Published:** 2023-12-25

**Authors:** Semra Ersöz, Anika Nissen, Reinhard Schütte

**Affiliations:** 1 Institute for Marketing and Retail Faculty of Economic Sciences University of Duisburg-Essen Essen Germany; 2 Institute for Business Administration Faculty of Economic Sciences University of Hagen Hagen Germany; 3 Institute for Computer Science and Business Information Systems Faculty of Economic Sciences University of Duisburg-Essen Essen Germany

**Keywords:** online pharmacy, emotion, facial expression, fNIRS, functional near-infrared spectroscopy, risk, trust, pharmacy, purchase, purchasing, consumer, consumers, customer, customers, drug, drugs, pharmaceutical, buy, buyer, pharmacies, perception, perceived, pharmaceutics, pharmaceutic, business, commerce, commercial, e-commerce

## Abstract

**Background:**

Online pharmacies are used less than other e-commerce sites in Germany. Shopping behavior does not correspond to consumption behavior, as online purchases are predominantly made for over-the-counter (OTC) medications.

**Objective:**

The objective of this study was to understand the purchasing experiences of online pharmacy customers in terms of critical factors for online pharmacy adoption.

**Methods:**

This study examined the perceived risk, perceived trust, and emotions related to purchasing medications online and, consequently, the purchase intention toward online pharmacies. In a within-subjects design (N=37 participants), 2 German online pharmacies with different perceptions of risk and trust were investigated for their main business, namely OTC and prescription drugs. The results of a preliminary study led to 1 online pharmacy with high and 1 with significantly low self-reported risk by the prestudy sample. Emotions were measured with a multimethod approach during and after the purchase situation as follows: (1) neural evaluation processes using functional near-infrared spectroscopy, (2) the automated direct motor response during the use of the online pharmacy via facial expression analysis (FaceReader), and (3) subjective evaluations through self-reports. Following the shopping experiences at both pharmacies for both product types, risk, trust, and purchase intention toward the pharmacies were assessed using self-assessments.

**Results:**

The 2 online pharmacies were rated differently in terms of risk, trust, emotions, and purchase intention. The high-risk pharmacy was also perceived as having lower trust and vice versa. Significantly stronger negative emotional expressions on customers’ faces and different neural activations in the ventromedial prefrontal cortex and dorsomedial prefrontal cortex were measured when purchasing prescription drugs from the high-risk pharmacy than from the low-risk pharmacy, combined with OTC medications. In line with this, customers’ self-ratings indicated higher negative emotions for the high-risk pharmacy and lower negative emotions for the low-risk pharmacy. Moreover, the ratings showed lower purchase intention for the high-risk pharmacy.

**Conclusions:**

Using multimethod measurements, we showed that the preceding neural activation and subsequent verbal evaluation of online pharmacies are reflected in the customers’ immediate emotional facial expressions. High-risk online pharmacies and prescription drugs lead to stronger negative emotional facial expressions and trigger neural evaluation processes that imply perceived loss. Low-risk online pharmacies and OTC medications lead to weaker negative emotional facial expressions and trigger neural evaluation processes that signify certainty and perceived reward. The results may provide an explanation for why OTC medications are purchased online more frequently than prescription medications.

## Introduction

The number of e-commerce websites and the resulting market are growing steadily [1], and so is the online pharmacy market [2,3]. Online sales of pharmaceuticals are experiencing tremendous growth worldwide and are expected to increase significantly in the next few years for the main business of pharmacies with over-the-counter (OTC) and prescription drugs [4]. Despite the great advantages of online shopping, such as convenience, special offers, service quality, and autonomy in shopping [5], statistics in Germany show that mainly OTC medications and less than 2% prescription medications are sold online. Sales in the OTC medication market amounted to €13 billion (US $14.2 billion) in 2022. Of that, around 22.6% was generated by online sales [6]. These figures do not match the typical consumption and demand for pharmaceuticals in Germany and indicate the particularly low acceptance of online pharmacies [7]. This is in stark contrast to the high acceptance of online shopping in general.

There are several factors that could contribute to the reluctance of consumers to purchase medications from online pharmacies. One possible explanation is the perceived higher risk associated with buying drugs online, which may be exacerbated by the growing presence of illicit pharmacies and counterfeit drugs on the market [8-10]. Additionally, consumers may be deterred by the lack of access to trained professionals who can provide information about medication effects [11,12]. Moreover, unlike most products sold online (eg, consumer electronics or fashion), counterfeit medications can have serious implications for a person’s physical and mental health [13].

Perceived risk is suggested to be powerful in explaining customer behavior because customers tend to avoid mistakes more than to maximize utility during purchasing [14-16]. It can be defined as a loss or negative consequence incurred by the consumer when purchasing a particular product and thereby depends on *what is acquired* and *how* or *where the acquisition takes place* [17-19]. Regarding the *what*, medications can be generally divided into (1) OTC medications, which can be purchased by one’s own decision, and (2) prescription medications, which should only be taken on the advice of a physician and therefore require a prescription (per German law §1 Arzneimittelverschreibungsverordnung [AMVV]). Regulations provide vital control mechanisms, as high-risk drugs require prescriptions, while less risky ones are available OTC, aligning with customers’ perceptions [20,21].

With regard to the *how* and *where*, e-commerce in general is a business model that provides an easy entry point to the market. A website can be constructed by a retailer to sell products with minimal regulations. Online pharmacies, in contrast, face stricter regulations with regard to setting up an online store and selling medications [22]. Although strict requirements prevail, one cannot speak of standardized ordering procedures that make shopping easier for customers.

Studies reveal that both the product and the pharmacy pose significant risks in regard to purchase intention in online pharmacies [23]. However, perceived risk must not be a barrier to adoption [24,25], but the lack of trust can be [25]. The willingness to take risks in a relationship can be understood as trust [26,27], and the question of whether a retailer is trustworthy plays a central role in the decision-making process of online pharmacy customers. If customers make the wrong decision, they may be at considerable risk, such as jeopardizing their health or even losing their lives [28]. Other research on online pharmacies has confirmed the negative influence of risk and the positive effect of trust on purchase intention [23,24,29-31]. Furthermore, trust perceptions were found to be negatively affected by perceived risk [29], while increased trust led to a decrease in the perceived risk associated with online pharmacies [31].

With regard to decisions under risk, social sciences, psychology, and economics have acknowledged that emotions play a key role in decision-making [32,33]. In this vein, several studies show that decisions can be predicted by immediate emotional states [34,35]. Komiak and Benbasat [36] argued that customers making trust decisions for products they cannot directly experience, such as medications, rely on spontaneous emotional processes that are less cognitively dominated. Several studies suggest that both cognitive and emotional processes are involved in perceived risk and perceived trust [37-41]. Emotions are proposed to guide decisions under risk, as demonstrated by the interdependence of emotions and risk in multiple works [38,41-44].

Perceived risk is typically associated with negative emotions [41,45,46]. Brain imaging studies also support the role of emotions in risk assessment, as damage to emotional processing regions can impair the ability to accurately assess risks [47-49]. Immediate emotional states have been found to significantly predict decisions, beyond anticipated emotions or subjective probabilities of risk outcomes [34,35,50].

In general, emotions serve several functions, including the evaluation of objects and events, the regulation of systems, the preparation and direction of action, the communication of reactions and behavioral intentions, and the monitoring of internal states and organism-environment interactions [51]. One of the most used definitions for emotions is the result of an extensive review of 92 different definitions [50]. According to this definition, emotion is “a complex set of interactions among subjective and objective factors, mediated by neural/hormonal systems, which can (a) give rise to affective experiences such as feelings of arousal, pleasure/displeasure; (b) generate cognitive processes such as emotionally relevant perceptual effects, appraisals, labeling processes; (c) activate widespread physiological adjustments to the arousing conditions; and (d) lead to behavior that is often, but not always, expressive, goal-directed, and adaptive” [50]. In line with this definition, Izard [52] proposed that emotions consist of neural circuits, response systems, and a feeling state or process that motivates and organizes cognition and action. Emotions are triggered by appraisal processes and expressed through immediate autonomic responses and reflected subjective feelings about the situation. These concepts are supported by a consensus among emotion researchers [53]. Therefore, perceived risk and perceived trust cues from pharmacy websites and products are thought to lead to evaluative processes and resulting emotional responses that can be measured through neural activity; immediate physiological responses, such as facial expressions; and reflected subjective feelings about the situation [52,54,55].

To understand the customers’ purchasing experience in online pharmacies, the overall goal of this paper is to investigate the emotional experience associated with the perceived risk and perceived trust of online pharmacies as an antecedent to behavioral intentions to purchase medication online. To achieve this goal, we used a multimethod approach, including the capture of customers’ self-reported perceptions, facial expressions, and neural appraisal processes. It is important to note that although facial expressions provide immediate and unfiltered emotional responses [56], verbalized subjective feelings can be prone to biases [44,57,58]. Therefore, the use of neural appraisal processes and facial expressions allowed us to capture emotional reactions that might not be visible in self-reported data.

## Methods

### Study Design

The study was designed as a within-subjects research and divided into 3 parts. In the first task, participants were asked to use the 2 prior selected online pharmacies, Apotal and DocMorris [59]*.* The online pharmacies were selected based on their different risk and trust ratings in a preliminary study. Both are legal, hold the European Union (EU) safety logo, and are listed in national mail order registers. As a search task, participants were provided with the scenario that they had met with an accident and were now limited in their movements. As a result, they now wanted to purchase their medications online. Participants were asked to buy well-known OTC painkillers (ibuprofen) and redeem the prescribed antithrombosis injection (Clexane). The task was completed once both medications were added to the shopping cart. The search task had to be executed on both online pharmacies; the order was randomized for each participant. During this task, participants’ faces were recorded via a video camera with a resolution of 1080 pixels and 30 frames per second, and facial expressions were later analyzed with FaceReader (Noldus) software.

After finishing their search task, participants were handed a questionnaire. First, a validated German version of the Positive and Negative Affect Scale (PANAS) was assessed for each included pharmacy to obtain insights into associated, self-reported emotional states related to the online pharmacy [60,61]. This was followed by questions on participants’ demographics. Finally, the experimenter measured the head circumference of participants, after which the third part of the study was started.

**Figure 1:**
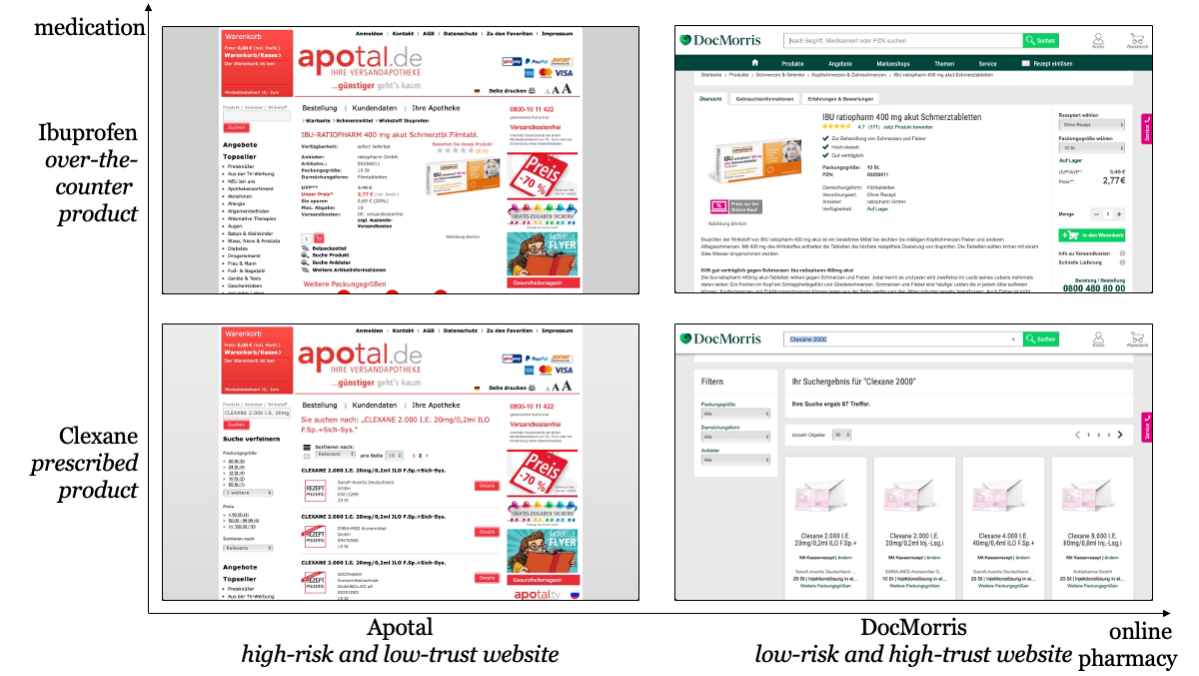
Stimuli used in the experimental task.

In the final part of the study, neural data were collected using the mobile neuroimaging method of functional near-infrared spectroscopy (fNIRS). In an event-related experimental paradigm, the online pharmacies were shown to participants as screenshots with the medications they had to search for in the first task. Each screenshot was shown for 4 seconds, after which a question from the scales for perceived risk (adapted from Forsythe and Shi [62]), perceived trust (adapted from Cyr et al [63]), and purchase intention (adapted from Gefen [64]) for the online pharmacies was shown with a 5-point Likert scale. Upon input from participants, a neutral cross randomly jittered between 2 and 4 seconds was shown. After that, the next screenshot was shown. Both online pharmacies were shown with both medications (Figure 1). This procedure continued until each question was asked for each screenshot. To ensure validity, the included scales in this study, except PANAS, were also used and validated in our preliminary study [59]*.*

After all tasks were completed and questions answered, the fNIRS headband was removed and participants were free to leave.

### Ethical Considerations

This study was reviewed by the Ethics Committee of the Medical Faculty of the University of Duisburg-Essen (approval number 21-9995-BO). Participants were informed about the study before the start of the study and were provided with an informed consent form. The recording and analysis of video data was pseudonymized. Only anonymized data were used in the publication. Participants received €10 (US $10.9) as compensation.

### Sample

We recruited 42 participants through a local university in September 2021. Exclusion criteria included existing neurological disease, reduced general health condition, no experience with online shopping, belonging to pharmaceutical staff, and taking medications with an effect on the central nervous system. Data from 5 (11.9%) participants were excluded: 1 participant because of a measurement error with FaceReader, 3 participants who did not correctly complete the search task on the online pharmacies, and 1 participant because of too much noise in the neural data. As a result, we included a final sample size of n=37 (88.1%) participants for further data analysis. Although this sample size might seem small for behavioral studies, it matched the average sample size of NeuroIS research, which was 38 participants [65]. Further, related fNIRS studies show that medium-large effects at 80% power can already be detected with a sample size of 20 participants [66]. Power analysis showed that with 37 participants, we had a power of 84.1% to effect sizes of |δ|>0.5 and a maximum type I error rate of 0.05.

The mean age was 28.6 (SD 9.52 years, min.=18 years, max.=62 years). Gender distribution was almost balanced, with 18 (48.6%) participants being female and 19 (51.4%) male. As handedness has been shown to potentially cause bias in neural activation in the prefrontal cortex (PFC), we assessed the handedness of participants using the laterality quotient (LQ) described by Salmaso and Longoni [67] (we used the German version of Götze [68]), which revealed that the majority of the sample was right-handed (n=33, 89.2%). The mean head circumference of participants was 55.5 (SD 2.73 cm). Most of the participants were employed (n=18, 48.6%), followed by a smaller number being university students (n=14, 37.8%) and school pupils (n=4, 10.8%), and 1 (2.7%) participant was searching for employment.

### Measurement Methods

#### Functional Near-Infrared Spectroscopy

It is widely acknowledged that fNIRS offers a mobile, robust, user-friendly, and nonintrusive method to assess the neural activity of participants at the cortical level [69-74]. Although neural correlates of emotion are frequently investigated in more primitive brain structures, such as the amygdala or the striatum, the appraisal processes that lead to an emotional experience are mainly processed in the PFC [75]. Several neuroimaging studies have found distinct neural patterns that might be related to different types of appraisals [76-79]. With regard to assessing risk and trust, however, it is primarily the medial parts of the PFC that come into play [48,49,80-82].

The fNIRS device sends near-infrared light into the brain at 2 (or more) wavelengths, which are reflected or absorbed by the hemoglobin in the blood [70,83]. Therefore, the levels of total hemoglobin, oxygenated as well as deoxygenated hemoglobin (HbO and HbR, respectively), in the brain regions under the fNIRS device are assessed [83,84]. In particular, HbO and HbR signals provide information about which brain region is activated to process the task or stimuli, because an increase in HbO levels and a decrease in HbR levels signifies neural activation of the given area [83].

##### Technical Specifications

**Figure 2:**
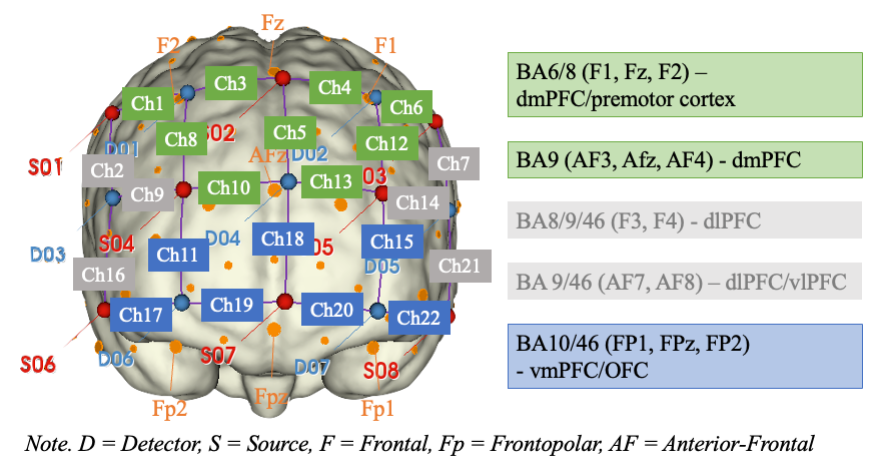
fNIRS montage design on the PFC. BA: Brodmann's Area; Ch: channel; dmPFC: dorsomedial prefrontal cortex; fNIRS: functional near-infrared spectroscopy; vmPFC; ventromedial prefrontal cortex.

The fNIRS device used in this study was a mobile, continuous-wave NIRSport 1 device developed by NIRX. The device comes with a sampling frequency of 7.81 Hz and has wavelengths set to 760 and 850 nm. For this study, measurements were focused on the PFC, which was covered with 8 sources, 7 long-distance detectors (LDDs; average distance set to 30 mm), and 8 short-distance detectors (SDDs; average distance set to 8 mm, 1 short distance detector for each source). SDDs are used to assess extracerebral activations in the fNIRS signal, and thus, they help filter out noise in the data and ensure that only neural activation is interpreted [85-87]. Overall, the fNIRS montage holds 22 channels (Chs) that cover most cerebral areas of the PFC, and which LDD Chs cover which brain region can be seen in Figure 2 (note that SDD Chs are not depicted in the figure).

##### Data Analysis

We analyzed raw fNIRS data with the Brain AnalyzIR Matlab toolbox [88]. First, the sampling frequency was resampled to 4 Hz to address the high autocorrelation in the fNIRS signal [89]. After that, we calculated the optical density, followed by correcting data with the included SDD Chs using linear minimum mean square estimations to filter out artifacts caused by respiration, the heart rate, Mayer waves, movements, and extracerebral activations [90,91]. Hemoglobin values (HbO and HbR) were calculated using the modified Beer-Lambert law with a partial path length factor of 0.10 [92,93]. Finally, effects on the subject level using an autoregressive model with iteratively reweighted least squares (AR-IRLS) algorithm were calculated for the generalized linear model (GLM).

#### Automated Facial Expression Analysis

The automated facial expression analysis (AFEA) with FaceReader software is derived from nonautomated analysis methods known as the facial action coding system (FACS) by Ekman and Friesen [94]. The approach is based on an understanding of facial anatomy in which observable facial muscle movements are classified according to a taxonomy or dictionary of sorts and the collective recognition of the movements is used for interpreting facial gestures as emotions [94-96].

##### Technical Specifications

FaceReader version 8.0 was used, which is able to detect 7 emotional states with their intensities: happy, neutral, sad, angry, disgust, surprise, and contempt. Intensity (inactive to active) ranges between 0 and 1. AFEA followed 4 steps: (1) *face finding*, in which the face is detected using the Viola-Jones algorithm; (2) *modeling*, in which over 500 key points of the face are used to model a 3D mask of the face by applying the active appearance method (AAM); (3) *classification*, in which the expression is aligned and classified using a trained artificial neural network; and (4) *deep face classification*, in which direct classification of image pixels is conducted to enhance the accuracy of the analysis. When AAM works insufficiently (eg, if parts of the face are hidden), an analysis of emotional states is carried out nonetheless. Another advantage is that identification or calibration is not required in order to start analysis [97,98].

##### Data Analysis

We analyzed the video recordings of participants with the face model “general,” which fits most people [98]. To estimate the best model fit, we ran the analysis with the maximum accuracy (slow) and frame by frame. In addition to the frequency of the facial expressions, we also scored the maximum intensities for the 7 types of facial expressions over time for each task (ibuprofen and Clexane) in each pharmacy (Apotal and DocMorris). We used the maximum intensities because of the quick onset and brief duration of milliseconds; using average intensities was not reasonable [99].

### Subjective Evaluation

The filtered responses of subjective feelings can be conceptualized along 2 major dimensions of valence (positive-negative) and activation. In line with this, Watson and Tellegen [100] proposed a consensual structure of emotion, where positive and negative dimensions of valence are combined with activation (inactive-active). Briefly, a positive affect (PA) reflects the extent of activation and enthusiasm, whereas a negative affect (NA) is the opposite of PA [100]. With this conceptualization, the conscious aspect of emotional experience becomes accessible, and emotional words that depend heavily on the receivers’ understanding [101] are reasonably clustered to avoid bias caused by the limitation of words. We used the PA-NA, which is one of the most applied measurement scales [102,103]. With regard to behavioral intentions, PA can be mapped to approach behavior, while NA can be mapped to avoidance behavior [104].

## Results

### Self-Reports

First, we tested for reliability (Cronbach α) of the used scales, which resulted in sufficient reliability (α>.75) for all scales (perceived risk α=.790; perceived trust α=.861; NA α=.847; use intention α=.883). Additionally, we calculated item-rest correlations to assess the internal consistency of the scales. Results showed that all included items had sufficient internal consistency (item-rest*>*0.3) and were therefore included in the analysis.

Second, to ensure successful risk and trust manipulations in this study, we analyzed self-reported results using repeated-measures univariate analysis of variance (rmANOVA), with the factors “online pharmacy” and “medications” as repeated measures and the constructs “perceived risk” and “perceived trust” as dependent variables. As all assumptions were met by the data and the assumption of sphericity was given, no corrections were made.

The results revealed significant differences in perceived risk (*F*_1,36_=92.809, *P*<.001, η^2^_p_=0.721) and perceived trust (*F*_1,36_=60.131, *P*<.001, η^2^_p_=0.626) between the 2 included pharmacies. Apotal was perceived to bear higher risk (mean 3.11, SD 0.117) and lower trust (mean 2.97, SD 0.154) compared to DocMorris. Perceived risk and perceived trust were not surveyed for medication types because we considered medication types as a secondary effect (the types of medications and the different procedures for purchasing the medications indicate different perceptions of risk). Further, we evaluated possible biases in online pharmacies’ ratings, since the screenshots showed the online pharmacies paired with the medications. No significant interaction effect between online pharmacy and medication (*F*_1,36_=0.026, *P*=.87, η^2^_p_=0.001) and trust (*F*_1,36_=0.661, *P*=.42, η^2^_p_=0.018) was found.

Testing the subjective feeling of emotion toward the online pharmacies with rmANOVA revealed a significantly higher NA (*F*_1,36_=52.485, *P*<.001, η^2^_p_=0.593) for Apotal (mean 1.96, SD 0.684) compared to DocMorris. Further, significantly higher use intentions were reported for DocMorris (mean 3.73, SD 0.156) compared to Apotal (*F*_1,36_=103.826, *P*≤.001, η^2^_p_=0.743). All means and SDs of the included self-reports are presented in Table 1.

**Table 1:** Results of the self-reports.

Online pharmacy	Perceived risk, mean (SD)	Perceived trust, mean (SD)	Use intention, mean (SD)	NA^a^, mean (SD)
Apotal	3.11 (0.12)	2.97 (0.15)	2.23 (0.16)	1.96 (0.68)
DocMorris	2.11 (0.09)	3.98 (0.09)	3.73 (0.12)	1.25 (0.30)

^a^NA: negative affect.

### Neural Activation

**Figure 3:**
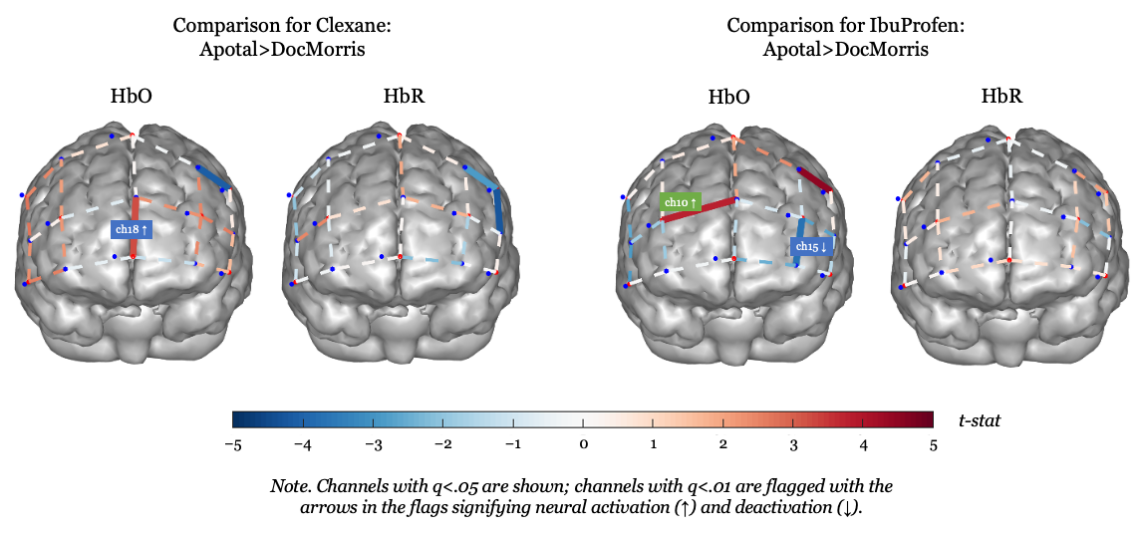
Comparison of higher-risk versus lower-risk online pharmacy (Apotal and DocMorris, respectively) for each medication product. Ch: channel; HbO: oxygenated hemoglobin; HbR: deoxygenated hemoglobin; q: false discovery rate (FDR)–corrected *P* value.

**Figure 4:**
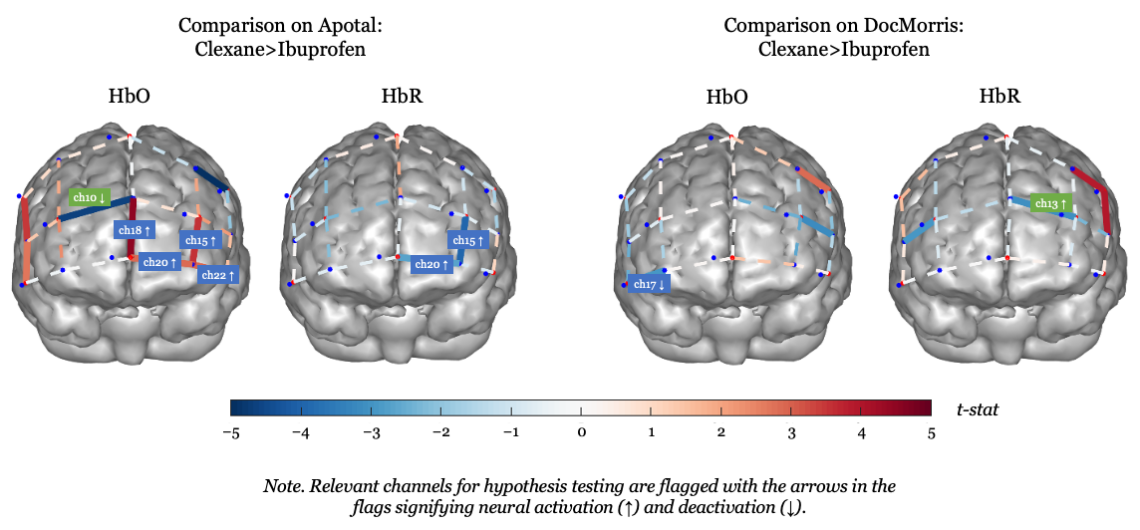
Comparison of prescription versus OTC medication (Clexane and ibuprofen, respectively) for each online pharmacy. Ch: channel; HbO: oxygenated hemoglobin; HbR: deoxygenated hemoglobin; q: false discovery rate (FDR)–corrected *P* value.

To analyze the neural data from fNIRS on a group level, we ran a mixed-effects model that used the 2 online pharmacies (Apotal and DocMorris) and the medications (ibuprofen and Clexane) as fixed effects and individual differences between participants as random effects. Further, it is important to note that an increase (red Ch) in HbO and a subsequent decrease in HbR (blue Ch) pointed to neural activation (see Figures 3 and 4 and Table 2). To avoid false positives, Chs in which both HbO and HbR showed a significant increase were omitted from further analysis (ie, Ch 6).

**Table 2:** Results of the mixed-effects model for comparison of the high-risk (Apotal) versus low-risk (DocMorris) online pharmacy for each medication.

Type	Ch^a^	Area	Condition	β	t_144_	q^b^	Power
HbO^c^	18	vmPFC^d^	Clexane (Apotal>DocMorris)	1.868	3.283	0.015	0.783
HbO	10	dmPFC^e^, right	Ibuprofen (Apotal>DocMorris)	1.489	3.813	0.003	0.904
HbO	15	vmPFC, left	Ibuprofen (Apotal>DocMorris)	–2.882	–3.742	0.004	0.892

^a^Ch: channel.

^b^q: false discovery rate (FDR)–corrected *P* value.

^c^HbO: oxygenated hemoglobin.

^d^vmPFC: ventromedial prefrontal cortex.

^e^dmPFC: dorsomedial prefrontal cortex.

#### Prescription Medication in the High-Risk Online Pharmacy

We identified significant activation of the ventromedial prefrontal cortex (vmPFC) only when the prescription medication (Clexane) was shown in the high-risk online pharmacy (Apotal). This became evident not only in the comparison between the 2 pharmacies when both showed Clexane (Figure 4, left, Ch 18 in HbO) but also when comparing the depiction of Clexane compared to ibuprofen both in Apotal (Figure 3, left, Chs 15, 18, 20, and 22 in HbO and Chs 15 and 20 in HbR; see also Table 3). Furthermore, only when comparing within the high-risk pharmacy’s website, we also identified deactivation of the right dorsomedial prefrontal cortex (dmPFC) when the prescription medication was shown compared to the OTC medication (Figure 3, left, Ch 10 in HbO).

**Table 3:** Results of the mixed-effects model for comparison of the prescription (Clexane) versus the OTC^a^ (ibuprofen) medication for each online pharmacy.

Type	Ch^b^	Area	Condition	β	t_144_	q^c^	Power
HbO^d^	10	dmPFC^e^, right	Apotal (Clexane>ibuprofen)	–1.698	–4.709	0.000	0.986
HbO	18	vmPFC^f^	Apotal (Clexane>ibuprofen)	2.606	4.557	0.001	0.979
HbO	15	vmPFC, left	Apotal (Clexane>ibuprofen)	2.732	3.598	0.006	0.863
HbR^g^	15	vmPFC, left	Apotal (Clexane>ibuprofen)	–1.950	–3.999	0.002	0.932
HbO	20	vmPFC, left	Apotal (Clexane>ibuprofen)	1.170	2.886	0.032	0.650
HbO	22	vmPFC, left	Apotal (Clexane>ibuprofen)	2.122	2.933	0.032	0.667
HbR	20	vmPFC, left	Apotal (Clexane>ibuprofen)	–0.604	–2.681	0.049	0.572
HbR	13	dmPFC, left	DocMorris (Clexane>ibuprofen)	–1.132	–3.087	0.024	0.721
HbO	17	vmPFC, right	DocMorris (Clexane>ibuprofen)	–1.593	–2.933	0.032	0.667

^a^OTC: over the counter.

^b^Ch: channel.

^c^q: false discovery rate (FDR)–corrected *P* value.

^d^HbO: oxygenated hemoglobin.

^e^dmPFC: dorsomedial prefrontal cortex.

^f^vmPFC: ventromedial prefrontal cortex.

^g^HbR: deoxygenated hemoglobin.

#### OTC Medication in the Low-Risk Online Pharmacy

We identified activation of the left vmPFC and deactivation of the right dmPFC when the OTC medication (ibuprofen) was depicted in the low-risk pharmacy (DocMorris) compared to the high-risk pharmacy (see Figure 4, right, Chs 15 and 10 in HbO). When comparing prescription and OTC medications within the low-risk pharmacy’s website, significant activation of the right vmPFC and deactivation of the left dmPFC were observed (Figure 3, right, Ch 13 in HbO and Ch 17 in HbR).

### Facial Expressions

Analogous to the neural results, we ran a mixed-effects model to analyze the results of the facial expression analysis on a group level related to the use of the online pharmacies (Apotal and DocMorris) and the medications (Clexane and ibuprofen) as fixed effects and individual differences between participants as random effects. The averages of the maximum intensities of negative facial expressions during the purchases were used for calculations.

We found significant differences in the intensity of negative emotions between the groups (*F*_3,108_=5.86, *P*<.001). These were identified between the pharmacies (*F*_1,108_=12.80, *P*<.001) and the medications (*F*_1,108_=4.74, *P*=.03). During the purchase at the high-risk pharmacy (Apotal), the intensity of negative emotions was higher (mean 0.310, SD 0.131) than during the purchase at the low-risk pharmacy (DocMorris; mean 0.283, SD 0.122). Additionally, during the purchase of the prescription medication (Clexane), the negative facial expression intensity was higher for both pharmacies (Apotal: mean 0.333, SD 0.145; DocMorris: mean 0.304, SD 0.142) than during the purchase of the OTC medication (ibuprofen; Apotal mean 0.287, SD 0.135; Docmorris mean 0.230, SD 0.123).

However, testing the differences between the medications in the 2 pharmacies revealed insignificant effects. Moreover, comparing the purchase of the prescription medication (Clexane) at the high-risk pharmacy (Apotal) with the same purchase at the low-risk pharmacy (DocMorris) showed no significant effect.

Comparing the purchase of the prescription medication (Clexane) at the high-risk pharmacy (Apotal) with the purchase of the OTC medication (ibuprofen) at the low-risk pharmacy (DocMorris) resulted in significantly higher intensities of negative facial expressions (t_108_=4.069, *P*<.001). All values are reported in Table 4.

**Table 4:** FaceReader results for the comparison of negative emotion intensities.

Comparison	Difference	SE	t_108_	*P* value
DocMorris vs Apotal	–0.027	0.0124	–2.177	.03
Ibuprofen vs Clexane	–0.044	0.0124	–3.577	<.001
Apotal (Clexane vs ibuprofen)	0.047	0.0175	2.661	.05
DocMorris (Clexane vs ibuprofen)	0.042	0.0175	2.398	.11
Clexane (Apotal vs DocMorris)	0.029	0.0175	1.671	.59
Ibuprofen (Apotal vs DocMorris)	0.025	0.0175	1.408	.97
Apotal and Clexane vs DocMorris and ibuprofen	0.071	0.0175	4.069	<.001

## Discussion

### Principal Findings

The subjective assessments of perceived risk and perceived trust confirmed that Apotal is perceived as having higher risk, lower trust, and more negative emotion than DocMorris. Similarly, the purchase intention is significantly lower for Apotal than for DocMorris. To obtain a deeper understanding of what leads to these self-reported ratings, we will take a closer look at the neural activity and facial expression results.

### Interpretation of Neural and Facial Expression Results

We identified consistent activation of the left vmPFC region when the prescription medication (Clexane) was combined with the high-risk online pharmacy (Apotal). This effect was further associated with the highest negative emotional intensity of facial expressions while using the 2 pharmacies’ websites. Our facial expression analysis further suggested that during the shopping process, the prescription medication had a more significant impact on negative emotional states than did the online pharmacy. A look at the related literature on the vmPFC’s functions and facial expression results provides us with further information about these findings.

In broader cognitive neuroscience, the vmPFC is frequently associated with value detection (ie, gain or loss) and with the regulation of (negative) emotion [105-108]. Furthermore, the significant role of this brain region in the evaluation of the potential implications of events for oneself was first stated in the somatic marker hypothesis and has been demonstrated in several studies that have shown that people with vmPFC damage are no longer able to make reasonable decisions [48,49,109]. The reason for this is that these persons lack the ability to accurately assess the risk (or potential loss) of a decision for themselves. This provides support for the somatic marker hypothesis that states that our decisions are framed and guided by emotions. Seeing the vmPFC activated for stimuli that are also associated with intense negative emotional facial expressions, and the higher self-rated perceived risk and NA, it is likely an indicator for negative emotional appraisal of medications and pharmacies’ websites.

The related literature that used AFEA revealed that negative emotions are often expressed more frequently and intensely than positive emotions [99,110,111]. It is further understood that risky situations evoke negative emotions [45,46], and in the case of online shopping, risk leads to a negative emotional experience due to the fear of potential loss [112]. Therefore, we propose the following first theoretical implication of our study:

Theoretical implication 1: High-risk online pharmacies and prescription medications lead to stronger negative emotional facial expressions and give rise to neural appraisal processes signifying perceived loss.

When looking at the OTC medication (ibuprofen) and the online pharmacy associated with high perceived trust and low perceived risk (DocMorris), the facial expression analysis underlines that the medication and the pharmacy are associated with significantly less negative emotional expressions. On the neural level, we further identified a different activation pattern. Although the vmPFC still showed significant activation, it was always accompanied by a significant deactivation of the dmPFC in the opposite hemisphere. Although the vmPFC has been related to processing the reward or loss value [113], areas of the dmPFC have been more associated with decision conflict [114]. In risky decision-making, the dmPFC has been found to be activated when information is incomplete and thus uncertainty is high [82,115]. Furthermore, high activity in the dmPFC has been found to be associated with risky choices [116,117], suggesting that dmPFC activation in risky behavior acts as a warning signal [82]. Given its *deactivation* when both the online pharmacy and the medication are perceived as lower in risk but not when one of them is perceived as higher in risk, this situation may be decoded as a certain and safe option. Due to the higher perceived trust and less negative emotional expressions, the vmPFC activation for this shopping situation may signify perceived reward. Therefore, the following second theoretical implication can be suggested:

Theoretical implication 2: Low-risk online pharmacies and OTC medications lead to weaker negative emotional facial expressions and give rise to neural appraisal processes signifying certainty and perceived reward.

### Practical Implications

Several consequences for industry can be derived from our theoretical implications. Given that the perceived risk of prescribed medications cannot be changed due to their nature, online pharmacies need to especially tackle trust-building mechanisms on their websites for the purchasing process of prescription and potentially high-risk medications. Based on our findings, there is potential to generate neural responses associated with certainty and reward for OTC medications, which could explain why these products are more commonly purchased online compared to prescription medications. One key difference between the presentation of prescription and OTC medications on the online pharmacy websites investigated is the lack of product images for prescription items: only an image of an envelope with the prescription or no image is displayed. It would be beneficial to include photos of the products themselves to aid in their identification. Additionally, it is important to provide detailed textual information about prescription medications, as the lack of such information may contribute to the negative emotional responses observed toward prescription medications, which indicates a higher risk perception toward them [20]. Studies confirm that customers refuse to buy medicines online, because they do not receive relevant information on the pharmacy websites. Customers are concerned that their health will be put at risk because they lack technical information, such as the expiration date, a release form, and the safety of the product for their health [10,11,13].

Regarding the process for placing prescription medication orders, both pharmacies investigated act according to German law (§17 Apothekenbetriebsordnung [ApoBetrO]). The customer sends the prescription via mail to obtain the medication. However, DocMorris displays the desired medication in the shopping basket and provides ordering instructions on both the product page and the shopping basket. In contrast, when attempting to add a prescription medication to the basket on the Apotal website, a new window containing a PDF file opens up. The file explains that customers must fill out a printed form and take it to the post office to obtain their medications. Notably, the desired medication does not appear in the shopping basket. Given that customers have to send their prescriptions and order via mail and cannot simply shop online is an obstacle to the perceived convenience of shopping for prescription medications online. Despite the availability of e-prescriptions in Germany since September 2022, the current lack of necessary technology in many doctors’ offices has resulted in conventional prescriptions remaining relevant [118]. In fact, it was estimated that conventional prescriptions would still be issued in 70% of cases in 2023 [119].

Therefore, adding trust-building elements to these product pages is of utmost importance for online pharmacies. Other than providing technical information, a further option would be to include certificates of trust on the website to ensure the authenticity of the pharmacy. Pharmacies can also leverage telemedicine and chats or telephone hotlines to provide personal advice to customers. In fact, our low-risk pharmacy already makes use of this and provides easy access to these services directly on the product page. According to a study in India [120], this would help purchases in online pharmacies. Finally, online pharmacies should ensure that the purchase process is as convenient as it can be under the given regulations, including the guarantee of product availability and timely delivery; information about both is essential and should be visible to the customer on the website. Evidence shows that online pharmacy customers wish for better logistics [121].

Neuroeconomic research has shown that establishing brands and having a high (positive) reputation can lead to neural “lock-in” effects [122,123]. The neural effects are also located in the vmPFC and signify an increased appraisal of reward value toward stores and products of the preferred brand. Therefore, establishing such effects in customers may neutralize the NA and perceived risk with ordering prescription medications online.

### Limitations and Future Research

We used a multimethod approach to gain deper insights into the appraisal processes and immediate emotional experience of online medication purchasing. However, this study has some major limitations that should be addressed by future research. First, we considered 2 operating online pharmacies in Germany and examples of prescription and OTC medications. Thereby, we may have a geological restriction in the generalization of our findings. Especially as restricting laws for pharmacies in Germany do not always apply to other countries, this research needs to be validated in other countries and cultures. Regarding the experimental design, we had to measure facial expressions and neural activity in 2 tasks of the study. That is because the fNIRS headband obscured parts of the face (eyebrows) that are essential for emotion recognition with FaceReader. Future research should particularly focus on making simultaneous measures of facial expressions and neural activity to validate our results during actual website use. Finally, by using 2 German online pharmacies that are among the top 10 in Germany [124], we can argue that the stimuli were not manipulated for the goals of the study but that we assessed the perceptions of risk and trust, including emotional experience, for a real-world problem. Due to the close-to-real-life situation, several biases can result from uncontrollable stimuli in the environment as well as from unpredictable effects from those stimuli. Here also, the importance of self-reports becomes evident in understanding cognitive concepts that are interconnected with emotions. Given this limitation, and to further derive rigorous design guidelines for prescription medication pages, future work may use controlled stimuli where different practical implications made in this paper are tested.

### Conclusion

For a health-related and underresearched specific e-commerce phenomenon, namely online pharmacies and their primary business of prescription and OTC medications, we examined perceived risk and perceived trust as critical antecedents for their adoption in the context of emotions in decision-making. Our results demonstrate that in the context of online pharmacies in particular, and e-commerce websites in general, deeper insights into the evaluation processes of websites and products will help us better understand how the risk and trust associated with them can influence emotions and thus behavioral intentions. Furthermore, we also investigated potential links between uncertainty and neural decision conflicts that stem from appraisal processes, and their relation to the perceived risk of online pharmacies, depending on the medications. Both facial expression analysis and neural results showed that several negative emotional attribution processes occur for prescription medications compared to OTC medications. Online pharmacies may therefore rethink how the product page of prescription medications should be designed so that the intense negative emotional experience, increased uncertainty, and perceived risk can be weakened for this product type. Building trust incentives into the product page and fostering a strong brand may elevate these negative effects of prescription medication and foster the success of online pharmacies.

## Abbreviations

AAM: active appearance method
AFEA: automated facial expression analysis
Ch: channel
dmPFC: dorsomedial prefrontal cortex
fNIRS: functional near-infrared spectroscopy
HbO: oxygenated hemoglobin
HbR: deoxygenated hemoglobin
LDD: long-distance detector
NA: negative affect
OTC: over the counter
PA: positive affect
PANAS: Positive and Negative Affect Scale
PFC: prefrontal cortex
SDD: short-distance detector
vmPFC: ventromedial prefrontal cortex

## Data Availability

All anonymized data collected for this paper as well as the data analyses are available on the Open Science Framework [125] website. Note that the video recordings for facial expression analysis are not shared as this would breach anonymization.
